# Transcriptome–Metabolome Combined Analysis of Central Carbon Metabolites in *Anoectochilus roxburghii* (Wall.) Lindl. Under Salt Stress

**DOI:** 10.3390/genes17050523

**Published:** 2026-04-29

**Authors:** Heping Li, Fangzhou Zhao, Huiming Huang, Shuhe Zhang, Jiangbo Lin, Debao Huang, Yimin Dai

**Affiliations:** 1 Institute of Subtropical Agriculture, Fujian Academy of Agricultural Sciences, Zhangzhou 363005, China; liheping@faas.cn (H.L.); huanghuiming@faas.cn (H.H.); zhangshuhe@faas.cn (S.Z.); linjiangbo@faas.cn (J.L.); 2 Key Laboratory of Institute of Industrial Crops, Institute of Industrial Crops, Shandong Academy of Agricultural Sciences, Jinan 250100, China; fangzhou@saas.ac.cn; 3 Department of Horticultural Science, North Carolina State University, Raleigh, NC 27607, USA

**Keywords:** *Anoectochilus roxburghii* (Wall.) Lindl., salt stress, central carbon metabolites, metabolomics, transcriptomics

## Abstract

**Background**: *Anoectochilus roxburghii* (Wall.) Lindl. is an endangered medicinal herb, and salt stress has been reported to promote the accumulation of bioactive secondary metabolites. Central carbon metabolism plays a key role in carbon allocation in plants; however, the integrated molecular and metabolic responses of *A. roxburghii* to salt stress remain largely unclear. **Method**: In this study, an integrated transcriptomic and metabolomic approach was employed to investigate the reprogramming of central carbon metabolism in *A. roxburghii* under 50, 100, and 200 mM NaCl treatments. **Results**: Metabolomic analysis revealed a significant accumulation of soluble sugars, which suggests enhanced osmotic adjustment and alteration in energy metabolism. Transcriptomic profiling identified 7019 upregulated and 5192 downregulated DEGs, with pathways related to the TCA cycle, galactose metabolism, and fructose/mannose metabolism predominantly upregulated, while oxidative phosphorylation was suppressed. Integrative transcriptome–metabolome profiling further identified key genes associated with oxaloacetate and fructose-6-phosphate, suggesting a coordinated regulation between central carbon metabolism and polysaccharide biosynthesis. **Conclusions**: Collectively, these findings demonstrate that salt stress induces coordinated metabolic and transcriptional reprogramming in *A. roxburghii*, driving carbon flux reallocation from growth-related processes toward osmoprotective metabolism. This provides a mechanistic basis for the enhancement of bioactive compounds under moderate stress and offers insights for improving both salt tolerance and medicinal quality in saline environments.

## 1. Introduction

*Anoectochilus roxburghii* (Wall.) Lindl. is a perennial herb plant widely distributed in subtropical regions of Asia and is highly valued for its medicinal properties [1,2]. Beyond its pharmacological applications, *A*. *roxburghii* also possesses ornamental value due to its elegant architecture, distinctive leaf morphology, and striking golden venation. Polysaccharides are recognized as key bioactive compounds in this plant, contributing significantly to its value in the medical nutrition industry with their diverse health-promoting functions [3,4]. Given the increasing demand for *A. roxburghii*, enhancing both its yield and medicinal quality has become a critical objective.

Understanding the molecular and metabolic responses of *A. roxburghii* to salinity stress is essential for optimizing its cultivation. Salinity is a major abiotic stress that severely limits plant growth, development, and productivity [5,6,7]. Excess salt disrupts water uptake and ion homeostasis, leading to osmotic stress, growth inhibition, and metabolic imbalance [8]. It also triggers complex physiological and biochemical responses, including oxidative stress resulting from the accumulation of reactive oxygen species (ROS) [9,10]. In plants, salt stress not only affects growth, development and physiological processes, but also plays a critical role in regulating the accumulation of bioactive compounds. Zhao et al. performed a combined analysis of wide-target metabolomics and transcriptomics, which revealed a significant accumulation of flavonoids and flavonol-like metabolites in the roots of flax under salt stress. Additionally, corresponding transcription factors were found to be involved in the biosynthesis pathways of flavonoids and flavonols [11]. The soluble phenolic compounds, anthocyanins and flavonoids in the salt-tolerant sugarcane strain CP-4333 were 2.5, 2.8 and 3.0 times those of the sensitive strain sugarcane strain HSF-240 [12]. This highlights the potential of controlled stress application as a strategy to improve the quality of medicinal herbs. Nevertheless, the current understanding of the primary and secondary metabolic responses, particularly those related to carbon metabolism, in *A. roxburghii* under salinity stress remains limited. As central carbon metabolism acts as a key hub linking primary and secondary metabolism, the molecular mechanisms by which it responds to salt stress and modulates secondary metabolism in this medicinal plant remain largely unknown. Therefore, systematically elucidating the coordinated regulation between central carbon metabolism and secondary metabolism under salt stress is crucial for uncovering the molecular basis of quality formation in *A. roxburghii* and guiding the production of high-quality medicinal materials.

Central carbon metabolism (CCM), encompassing glycolysis, the tricarboxylic acid cycle (TCA), and the pentose phosphate pathway, is essential for plant growth, development, and responses to environmental stressors [13]. For instance, environmental stresses such as drought, flooding, and salinity can trigger substantial fluctuations in central carbon metabolites, thereby reshaping the global metabolic network [14,15,16]. Sharma et al. [15] demonstrated that arbuscular mycorrhizal fungi ameliorate salt stress by modulating CCM, promoting the biosynthesis of stress-mitigating metabolites (e.g., γ-aminobutyric acid [GABA], malic acid) and bypassing salt-sensitive enzymatic steps in the TCA cycle. In addition to providing energy and carbon skeletons for cellular maintenance and growth, CCM supplies key precursors for both primary and specialized metabolites that are essential for plant stress responses [17,18,19]. This close linkage between central carbon metabolism and secondary metabolites biosynthesis underscores the importance of understanding CCM reprogramming under environmental stress [20,21].

Given the increasing importance of *A. roxburghii*, there is a pressing need to elucidate the molecular mechanisms underlying its response to salt stress. Integrated transcriptomic and metabolomic analyses provide a powerful approach to uncover the coordinated gene regulatory networks and metabolic pathways associated with stress adaptation. However, how these stress-responsive processes are coordinated with central carbon metabolism, particularly in terms of carbon allocation and metabolic reprogramming, remains poorly understood in *A. roxburghii.* Therefore, identifying key genes, metabolites, and pathways involved in central carbon metabolism is essential for understanding salt tolerance mechanisms and for developing strategies to improve both the yield and medicinal quality of *A. roxburghii* in saline environments. This study aims to investigate the coordinated transcriptional and metabolic reprogramming of central carbon metabolism in *A*. *roxburghii* under salt stress, in order to elucidate the molecular basis of medicinal quality formation.

Despite its medicinal and ornamental value, the integrated molecular and metabolic responses of *A. roxburghii* to salt stress remain largely unexplored [22]. In particular, the coordinated regulation between transcriptomic changes and metabolite accumulation in central carbon pathways under salinity remains poorly understood. To address this question, the present study employs a combined transcriptome and metabolome analysis to investigate the responses of CCM in *A. roxburghii* under salinity stress. It was hypothesized that salt stress triggers coordinated transcriptional and metabolic reprogramming of CCM, redirecting carbon flux from growth-related processes toward osmotic adjustment and secondary metabolism, thus establishing a metabolic foundation for stress adaptation and potential enhancement of bioactive compound accumulation.

## 2. Materials and Methods

### 2.1. Plant Growth and Salt Treatment

Plant materials were provided from the Institute of Subtropical Agriculture, Fujian Academy of Agricultural Sciences. The plants were propagated via tissue culture and grown in a greenhouse for three months under controlled shade conditions to minimize environmental stress. A control group irrigated with distilled water and three treatment groups irrigated with NaCl concentrations of 50, 100, and 200 mM were selected to establish a gradient from moderate physiological stress, which may elicit adaptive or stimulatory responses, to severe growth-inhibitory stress, as commonly applied in studies investigating salt tolerance mechanisms in plants [23]. Fresh leaf samples were collected from plants treated with 0, 50, 100, or 200 mM NaCl (Xilong Scientific, Foshan, China) for one month, immediately flash-frozen in liquid nitrogen, and stored at −80 °C for subsequent metabolomic and transcriptomic analyses. For each treatment, three independent biological replicates were analyzed. Each replicate consisted of a pool of leaf tissue harvested from five randomly selected plants to minimize individual variation.

### 2.2. Chlorophyll Fluorescence Measured

Chlorophyll fluorescence parameters were measured using the portable fluorometer FluorPen 110 (Photon Systems Instruments, Drásov, Czech Republic). For each treatment, measurements were taken on the second to third fully expanded leaves below the shoot apex from ten uniformly grown plants. Real-time chlorophyll fluorescence Ft and a measure of photosystem II (PSII) efficiency quantum yield (QY = Fv′/Fm′) under light adaptation were determined.

### 2.3. Sample Extraction and UPLC Conditions

The freeze-dried leaves were ground into a fine powder, and 0.1 g of each sample was transferred into 2 mL microcentrifuge tubes. To extract metabolites, 0.6 mL of acetonitrile was added, followed by vortexing for 1 min and ultrasonic treatment at 30 °C for 30 min. The samples were then centrifuged at 4000 rpm for 10 min, and 300 μL of the resulting supernatant was transferred to a 1.5 mL microcentrifuge tube. Subsequently, 150 μL of 200 mM 3-nitrophenylhydrazine (3-NPH) and 150 μL of 120 mM 1-ethyl-3-(3-dimethylaminopropyl) carbodiimide were added. After vortexing for 1 min, the reaction was carried out at 40 °C for 1 h. The mixture was then centrifuged at 12,000 rpm for 15 min at 4 °C. The final supernatant was filtered through a 0.22 μm membrane filter and subjected to analysis using a liquid chromatography-tandem mass spectrometry (LC-MS/MS) system (AB SCIEX 5500 Qtrap–MS, Framingham, MA, USA). Chromatographic separation was conducted using a Waters Acquity Ultra Performance LC (UPLC, Milford, MA, USA) equipped with an ACQUITY UPLC HSS T3 column (1.8 μm pore size, 2.1 × 100 mm). The mobile phase consisted of solvent A (water) and solvent B (methanol). A 6 μL aliquot of each sample was injected, and the analysis was carried out at 35 °C with a flow rate of 0.3 mL/min. The gradient program was as follows: isocratic at 60% B (A: 40% acetonitrile, B: 60% methanol) from 0 to 2 min, followed by a decrease to 5% A and 95% B from 2 to 6 min, and then re-equilibration at 60% A and 40% B until the end of the run. The mass spectrometric conditions were as follows: ion source, ESI; curtain gas, 35 arb; collision gas, 7 arb; ion spray voltage, 4500 V; ion source temperature, 450 °C; ion source gases, 55 arb each for Ion Source Gas1 and Ion Source Gas2. The MRM acquisition parameters were established based on the aforementioned chromatographic and mass spectrometric conditions, and standard solution preparations were injected into the sample vials for analysis. Quality control was performed by analyzing pooled sample extracts (QC samples) at regular intervals throughout the analytical sequence to monitor instrument stability. Raw data were processed using MultiQuant Software (Version 3.0.2) for peak integration, alignment, and quantification. The metabolite content was calculated using the standard curve.

### 2.4. RNA-Seq Analysis and Identification of Differentially Expressed Genes

The total RNA extraction, cDNA library construction, and transcriptome sequencing were performed by Gene Denovo Biotechnology Co., Ltd. (Guangzhou, China). RNA was isolated using Trizol reagent (Life technologies, Waltham, MA, USA), and its quality and integrity were assessed prior to downstream processing. The cDNA library was constructed using the Hieff NGS@ Ultima Dual-mode mRNA library Prep kit (Takara Bio Inc., Shiga, Japan) according to the manufacturer’s instructions. Since no reference genome was available for *A. roxburghii* at the project initiation, RSEM (Version 1.3.1) [24] was employed to quantify the isoforms obtained from third-generation sequencing assembly. RSEM estimates read counts and transcript abundances using a generative model of RNA-seq reads and the Expectation-Maximization (EM) algorithm. Transcript expression levels were quantified using the fragments per kilobase of transcript per million mapped reads (FPKM), as described by Mortazavi et al. [25]. Subsequently, gene function annotation and structural characterization were performed. The BLASTX (Version 2.15.0) program (http://www.ncbi.nlm.nih.gov/BLAST/, accessed on 5 September 2023) was employed to align transcript sequences against public databases, including NR, Swiss-Prot, KEGG, and COG databases with an E-value threshold of 1 × 10^−5^.

Differentially expressed genes were identified with the DESeq2 (Version 1.36.0) package, using thresholds of false discovery rate (FDR) below 0.05 and absolute fold change ≥ 2 [26,27]. Gene Ontology (GO) and Kyoto Encyclopedia of Genes and Genomes (KEGG) annotations were used to determine the function of DEGs. GO and KEGG enrichment analyses were performed using the OmicShare platform (https://www.omicshare.com/tools, accessed on 5 September 2023).

### 2.5. Correlation Analysis Between Metabolomics and Transcriptomics

Based on gene expression levels and metabolite abundances, an intersection of differentially expressed genes (DEGs) and differentially accumulated metabolites (DAMs) was obtained for the three comparison groups (CK-vs-I, CK-vs-II, and CK-vs-III, where CK denotes the control group (0 mM NaCl), and I, II, III represent the 50, 100, and 200 mM NaCl treatment groups, respectively). The correlation coefficients between the DEGs and DAMs in these pathways were calculated.

Pearson correlation coefficients were calculated to assess pairwise associations between the expression levels of DEGs and the abundance of DAMs. A stringent threshold of absolute correlation coefficient |r| > 0.9 with a significance level of *p* < 0.01 was applied to identify the strongest and most statistically significant associations. This high threshold was chosen to reduce network complexity and focus on gene-metabolite relationships with the highest potential biological relevance for subsequent regulatory network analysis. For genes with the same symbol, the pair with the highest absolute correlation coefficient was retained. A gene-metabolite correlation network was then constructed to visualize these high-confidence relationships.

### 2.6. qRT-PCR Analysis

Total RNA was extracted from leaf samples using a Plant Total RNA extraction Kit (Zomanbio, Beijing, China), according to the manufacturer’s instructions, and stored at −80 °C. Genomic DNA in RNA samples was removed by treatment with the RNase-free DNase I (TaKaRa, Kusatsu, Japan) following the manufacturer’s instructions. First-strand cDNA was synthesized from total RNA (≈200 ng) in a 20 μL volume using Evo M-MLV RT Premix for qPCR (Accurate Biology, Changsha, China), according to the instructions. Actin was used as a reference gene. All primers were synthesized in Sangon Co., Ltd. (Shanghai, China). The cDNA from samples treated with different salt concentrations was used as a template, diluted to 50 ng/μL, and subjected to qPCR analysis using the LightCycler 96 fluorescence quantitative PCR instrument (Roche, Mannheim, Germany). The PCR reaction system (20 μL) included 10 μL of 2 × SYBR Green Pro Taq HS Premix (Accurate Biology, Changsha, China), 1 μL of template cDNA, 0.5 μL each of 10 μmol/L forward and reverse primers (Table 1), and 8 μL of ddH_2_O. The PCR program comprised an initial denaturation at 95 °C for 30 s, followed by 50 cycles of denaturation at 95 °C for 10 s, annealing and extension at 60 °C for 20 s. Three replicates were set for each sample, and gene relative expression levels were calculated using the 2^−ΔΔCt^ method [28].

### 2.7. Statistical Analysis

For physiological and individual metabolite data, significance was assessed using one-way ANOVA followed by Tukey’s HSD post hoc test, after verifying assumptions of normality (Shapiro–Wilk test) and homogeneity of variances (Levene’s test). For data violating these assumptions, the non-parametric Kruskal–Wallis test with Dunn’s post hoc test was used.

For transcriptomics, differentially expressed genes (DEGs) were identified using DESeq2 with an FDR-adjusted *p*-value (padj) < 0.05 and |log_2_FoldChange| ≥ 2. For metabolomics, differentially accumulated metabolites (DAMs) were identified based on a Variable Importance in Projection (VIP) score > 1.0 from OPLS-DA models and a *p*-value < 0.05 from univariate tests, with false discovery rate (FDR) correction applied.

Statistical comparisons between control and treatment groups were assessed using one-way (ANOVA) or non-parametric test as appropriate, performed with IBM SPSS Statistics 26 (IBM Corp., Armonk, NY, USA). Significance levels were defined as *p* < 0.05. Data visualization and plotting were conducted using Origin 2021 (OriginLab Corporation, Northampton, MA, USA).

## 3. Results

### 3.1. Growth and the PSII Quantum Yield of A. roxburghii Plants Under Salt Stress

**Figure 1:**
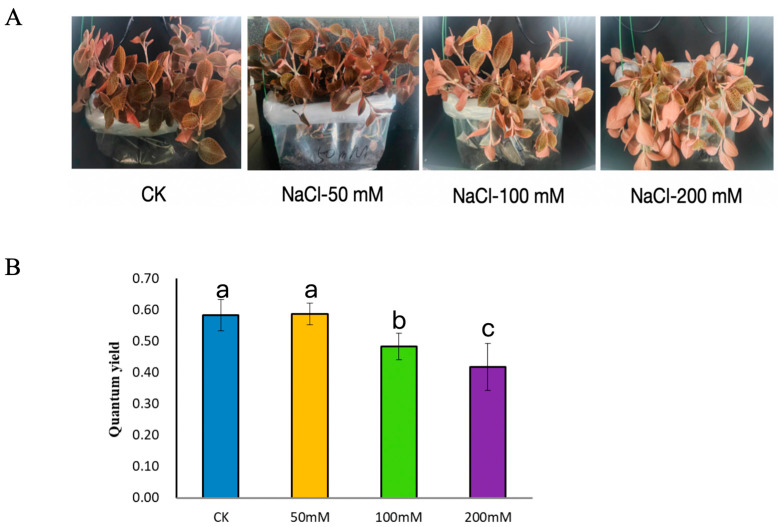
Morphological characteristics (**A**) and Chlorophyll fluorescence parameters (**B**) of *A. roxburghii* under different concentrations of NaCl. CK: Control (distilled water, 0 mM NaCl). Different lowercase letters indicate different significant differences (*p* < 0.05).

Under different concentrations of NaCl salt stress, the growth status of *A. roxburghii* gradually deteriorated with increasing salt concentration: 50 mM NaCl treatment had a slight effect on its growth; leaves showed wilting and abnormal coloration under 100 mM treatment; and the plants exhibited severe growth weakness under 200 mM treatment (Figure 1A). Meanwhile, the quantum yield (QY) decreased significantly with the intensification of salt stress: 50 mM NaCl treatment had no significant effect on QY, while 100 mM and 200 mM treatments reduced PSII efficiency by approximately 15% and 30%, respectively, compared to the control, indicating that high salt stress severely inhibits the photosynthetic system function of *A. roxburghii* (Figure 1B).

### 3.2. Metabolome Analysis of A. roxburghii Under Salt Stress

**Figure 2:**
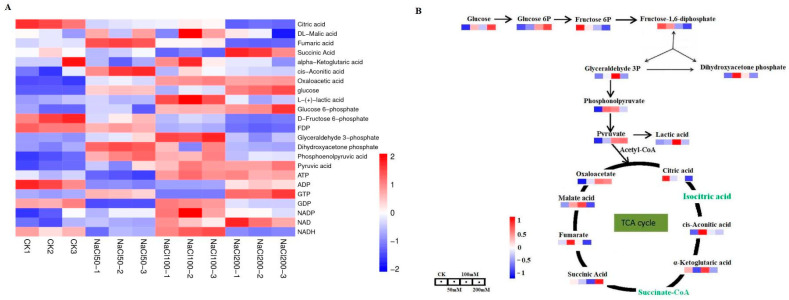
Analysis of central carbon metabolites in leaves of *A. roxburghii* under salt stress. (**A**) Heatmap of differentially accumulated metabolites (DAMs). Rows represent metabolites, columns represent treatment groups. Red indicates increased abundance, blue indicates decreased abundance. (**B**) Schematic of the central carbon metabolic network. Metabolite nodes are colored according to their measured change: red for increase, blue for decrease, and green for metabolites that were not detected in this study.

In order to analyze the changes in different metabolites under salt treatment at different time points, liquid chromatography-tandem mass spectrometry (LC-MS/MS) was used to determine the status of these metabolites. A total of 27 metabolites involved in central carbon metabolism were selected for further analysis. In general, a total of 23 of 29 selected metabolites were detected (Figure 2A; Table S1), whereas succinate-CoA, isocitric acid, 2,3-diphosphoglyceric acid, 3-phosphoglyceric acid, 2-phosphoglyceric acid, and NADPH were not detected. Following NaCl treatment, oxaloacetate, glucose, glucose-6-phosphate, pyruvate, and phosphoenolpyruvate were significantly increased (|log_2_FC| > 1 and *p* < 0.05), whereas citrate, fructose-6-phosphate, and fructose-1,6-bisphosphate were significantly decreased (|log_2_FC| > 1 and *p* < 0.05) (Figure 2; Table S1). The significant increase in soluble sugars such as glucose and glucose-6-phosphate can reduce the osmotic potential of cells, enhance water uptake by cells, and alleviate the damage caused by salt stress. In terms of energy metabolism, the ADP/ATP ratio did not differ significantly between the 50 mM NaCl treatment group and control group, whereas 100 mM and 200 mM NaCl treatments induced a significant change in the ADP/ATP ratio relative to the control—specifically, an increase in ATP abundance and a reduction in ADP levels (Table S1). This finding suggests that NaCl stress slows the rate of oxidative phosphorylation in cells, affecting the cell’s energy supply.

### 3.3. Transcriptome Analysis of A. roxburghii Plants Under Salt Stress

**Figure 3:**
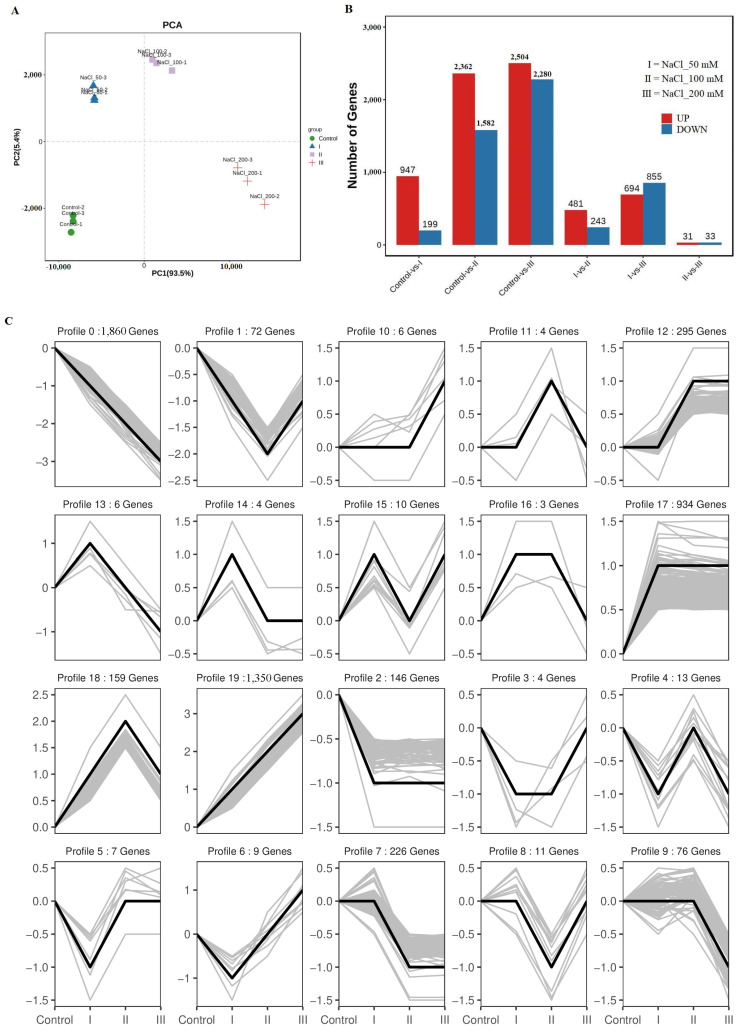
Profiles of transcriptomics. (**A**) PCA of transcriptome data. The X-axis represents PC1, the Y-axis represents PC2, and different samples are distinguished by different colors. (**B**) Statistical analysis of DEGs in all comparisons. (**C**) Trend analysis of differentially expressed genes.

To examine the effect of salt stress on central carbon metabolism in *A. roxburghii*, transcriptome sequencing was performed. The transcriptome profiles were then subjected to principal component analysis (PCA), and the PCA score plots exhibited an obvious separation between four NaCl (0, 50, 100, and 200 mM) treatments. The first principal component (PC1) was 84.19% and the second principal component (PC2) were 6.03% (Figure 3A).

This yielded 75,817,913 bp of high-quality clean reads, which were assembled into 30,087 transcripts with an average length of 2520.21 bp and an N50 value of 2798 bp. The average Q30 score was >92%, and the average mapping rate was >82% (Table 2). Additionally, RNA integrity was verified for all samples prior to library construction, with RNA Integrity Numbers (RIN) > 7, ensuring high-quality input material. These transcripts were subsequently annotated by alignment against major public databases, including the NCBI non-redundant protein (Nr) database, Swiss-Prot, KEGG, and KOG. In total, 29,087 isoforms were successfully annotated in at least one public database, including 29,066 hits in the Nr database, 28,871 in the KEGG database, and 25,004 in the Swiss-Prot database.

Differentially expressed genes (DEGs) were identified using DESeq2, resulting in a total of 12,211 DEGs, including 7019 upregulated and 5192 downregulated genes (Figure 3B). The comparison between the 200 mM NaCl treatment group and the control group yielded the highest number of DEGs (4784), followed by the 100 mM vs. control comparison (3944 DEGs) (Figure 3B). Notably, only 64 DEGs were identified in the 100 mM vs. 200 mM NaCl comparison, which suggests that the transcriptional response to NaCl stress reaches a saturation point at concentrations ≥ 100 mM, with minimal additional changes in gene expression at higher salt levels (Figure 3B). Trend analysis of all DEGs across the salt stress gradient revealed distinct expression patterns (Figure 3C). The largest cluster, comprising 1860 DEGs, exhibited a progressive down-regulation pattern as salt concentration increased. The second-largest cluster, containing 1356 DEGs, showed a corresponding up-regulation pattern.

**Figure 4:**
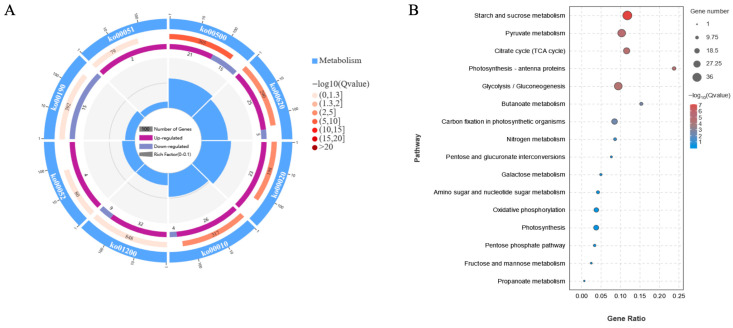
The KEGG pathway enrichment analyses performed on DEGs mapped to eight KEGG pathways associated with central carbon metabolism using cycle plot (**A**) and bubble plots (**B**).

To further elucidate the effects of salt stress on central carbon metabolism, enrichment analysis was conducted on DEGs mapped to eight KEGG pathways associated with central carbon metabolism: carbon metabolism, glycolysis/gluconeogenesis, tricarboxylic acid cycle (TCA cycle), starch and sucrose metabolism, galactose metabolism, fructose and mannose metabolism, pyruvate metabolism, and oxidative phosphorylation (Figure 4). The results showed that all DEGs mapped in the TCA cycle (Ko00020), galactose metabolism (Ko00052), and fructose and mannose metabolism (Ko00051) pathways were upregulated; most DEGs related to pyruvate metabolism (Ko00620), glycolysis/gluconeogenesis (Ko00010), and carbon metabolism (Ko01200) were also upregulated; however, all DEGs associated with oxidative phosphorylation (Ko00190) were downregulated (Figure 4A). Starch and sucrose metabolism (Ko00500), TCA cycle (Ko00020), and pyruvate metabolism (Ko00620) exhibited the highest RichFactor values, indicating that these pathways were the most heavily impacted by NaCl stress at the transcriptional level (Figure 4B).

### 3.4. Integrated Transcriptome–Metabolome Analysis of DEGs and DAMs in Response to Salt Stress

**Figure 5:**
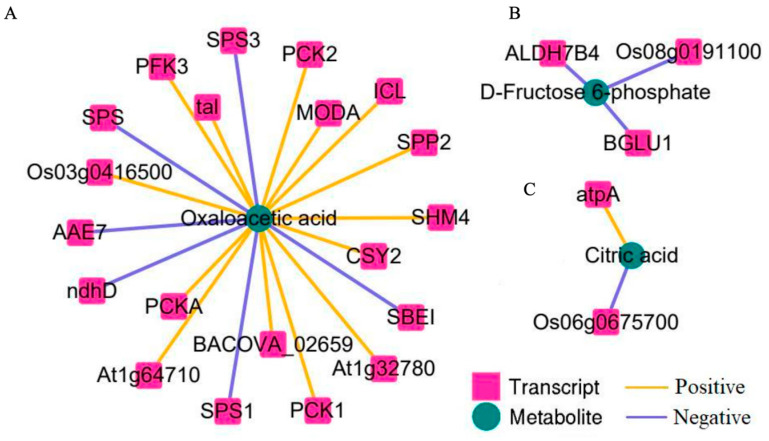
Connection network between DEGs and DAMs. DAMs: (**A**) Oxaloacetic acid; (**B**) D-Fructose 6-phosphate; (**C**) Citric acid. Note: Pink boxes represent genes, green diamonds represent metabolites. Yellow lines indicate positive correlation, purple lines indicate negative correlation.

Pearson correlation coefficients were computed to assess associations between the identified DEGs and DAMs. To identify the strongest and most statistically significant relationships, a stringent threshold (absolute correlation coefficient |r| ≥ 0.9, *p* < 0.01) was applied. Gene–metabolite pairs meeting this threshold were used to construct a correlation network (Figure 5). Within this network, oxaloacetate levels were significantly correlated with 20 DEGs, of which 14 showed positive and 6 showed negative correlations. The positively correlated genes were primarily annotated to central carbon metabolic pathways, including phosphoenolpyruvate carboxykinase (*PCKA*, *PCK1*, and *PCK2*), phosphofructokinase (*PFK3*), sucrose-phosphatase (*SPP2*), malic enzyme (*MODA*), and citrate synthase (*CSY2*) (Figure 5A). In contrast, six DEGs, including sucrose phosphate synthase (*SPS*, *SPS1*, and *SPS3*) and starch branching enzyme (*SBEI*), were negatively correlated with oxaloacetate (Figure 5A). Additionally, three DEGs were negatively correlated with fructose-6-phosphate, while two genes were associated with citrate levels, one positively and one negatively (Figure 5B,C).

### 3.5. qRT-PCR Validation of Key DEGs Involved in Central Carbon Metabolism

To validate the expression of genes associated with central carbon metabolism identified through transcriptome sequencing under salt stress in *A. roxburghii*, 12 key genes were chosen for quantitative real-time PCR (qRT-PCR) analysis. These genes are functionally related to the TCA cycle, starch biosynthesis, cellulose biosynthesis, sucrose metabolism, and oxidative phosphorylation. The relative expression levels of these genes were calculated using actin as a standard. The qRT-PCR results showed expression patterns consistent with the RNA-seq data, thereby confirming the reliability and robustness of the transcriptomic analysis under salt stress conditions.

**Figure 6:**
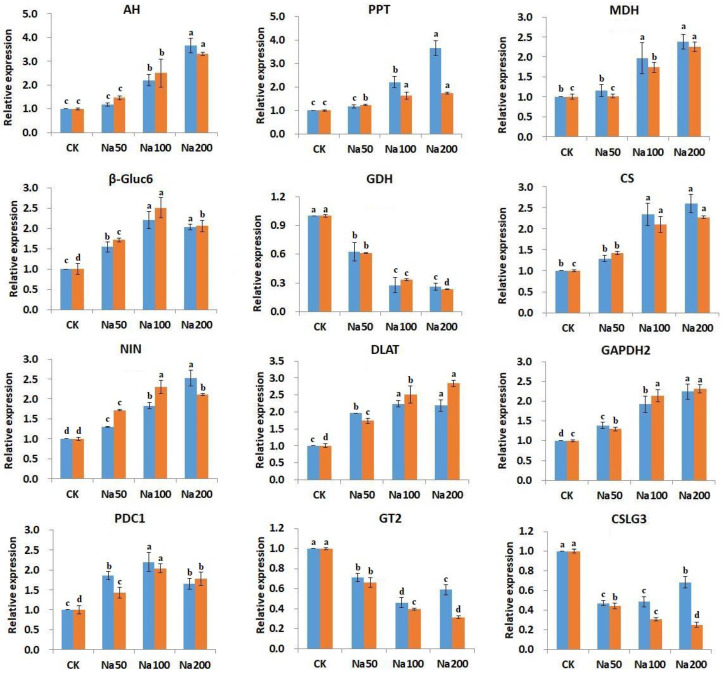
qRT-PCR validation of 12 key DEGs involved in central carbon metabolism. Gene abbreviations and corresponding full names are as follows: AH: aconitate hydratase 3; PPT: phosphoenolpyruvate/phosphate translocator; MDH: malate dehydrogenase; β-Gluc6: beta-glucosidase 6; GDH: glycerate dehydrogenase; CS: citrate synthase; NIN: alkaline/neutral invertase; DLAT: dihydrolipoyllysine-residue acetyltransferase component 2; GAPDH2: glyceraldehyde-3-phosphate dehydrogenase 2; PDC1: pyruvate decarboxylase 1; GT2: glycosyltransferase 2; CSG3: cellulose synthase-like protein G3. Blue represents the qRT-PCR results, while orange indicates the RNA-seq sequencing results (FPKM). Error bars represent mean ± standard deviation (*n* = 3). Different lowercase letters indicate different significant differences (*p* < 0.05).

The qRT-PCR results (Figure 6) showed that the genes encoding glycerate dehydrogenase, cellulose synthase, and glycosyltransferases were down-regulated under salt treatment, whereas the remaining nine genes were up-regulated. These findings suggest that salt stress inhibits cellulose and polysaccharide biosynthesis while activating genes associated with the TCA cycle and promoting central carbon metabolism. The accumulation of monosaccharides may play a critical regulatory role in osmotic adjustment in response to salt-induced cellular stress.

## 4. Discussion

Salinity stress is a major environmental constraint that severely limits plant growth, development, and agricultural productivity. In high-value medicinal plants such as *A. roxburghii*, understanding the physiological and molecular adaptations to salt stress is crucial not only for improving cultivation resilience but also for enhancing the biosynthesis of bioactive secondary metabolites through controlled stress application. Our integrated transcriptomic and metabolomic analyses reveal that salt stress induces a coordinated reprogramming of central carbon metabolism in *A. roxburghii*, resulting in a strategic reallocation of carbon flux from growth-related processes toward osmotic adjustment and secondary metabolite production.

### 4.1. Metabolic Reconfiguration Towards Osmotic Adjustment and Energy Remodeling

Our metabolomic data demonstrate that salt stress induces significant alterations in central carbon metabolites, reflecting a reprogramming of carbon flux under stress conditions. The marked accumulation of soluble sugars, such as glucose and glucose-6-phosphate, represents a typical osmotic adjustment response, enabling plants to maintain cellular turgor and water homeostasis under salinity stress [29,30]. This accumulation of osmotically active solutes supports stress tolerance by mitigating dehydration and stabilizing cellular functions. In parallel, the observed increase in ATP levels accompanied by a reduced ADP/ATP ratio suggests an imbalance in mitochondrial energy metabolism, likely associated with impaired oxidative phosphorylation efficiency. This indicates that severe salt stress disrupts the mitochondrial electron transport chain, leading to a reconfiguration of energy metabolism, as reported in other plant species under similar stress conditions [31]. Notably, the decrease in glycolytic intermediates such as fructose-1,6-bisphosphate suggests a shift in glycolytic flux, potentially redirecting carbon toward osmoprotective metabolite accumulation and other stress-responsive pathways. Together, these results support a model in which salt stress drives carbon flux reallocation from growth-related metabolism toward osmotic adjustment and stress adaptation.

### 4.2. Transcriptional Reprogramming Underpins the Metabolic Shift

Global transcriptome analysis provided a molecular basis for the observed metabolic reprogramming under salt stress. The widespread differential expression of genes involved in glycolysis/gluconeogenesis, the TCA cycle, and pyruvate metabolism suggests a coordinated transcriptional response to sustain energy (ATP) and reducing power (NAD(P)H) production, as well as to supply carbon skeletons for the biosynthesis of compatible solutes and antioxidants. The coordinated up-regulation of genes associated with the TCA cycle and anaplerotic reactions (e.g., those replenishing oxaloacetate) reflects an increased demand for metabolic intermediates, supporting enhanced carbon flux through central metabolic pathways under stress conditions [32]. In contrast, the downregulation of genes involved in oxidative phosphorylation is consistent with metabolomic evidence of impaired mitochondrial energy transduction, indicating a shift in energy metabolism under severe salinity stress [31]. Notably, the apparent saturation of transcriptional response at 100 mM NaCl, with limited additional changes at higher concentrations, suggests the activation of a core adaptive program up to a cellular threshold, beyond which cellular homeostasis is compromised [33]. Together, these findings indicate that transcriptional reprogramming acts as a regulatory layer driving carbon flux reallocation and metabolic adjustment under salt stress.

### 4.3. Integrated Analysis Reveals a Regulatory Network Prioritizing Defense over Growth

The integrated gene-metabolite correlation network revealed significant associations (|r| > 0.9, *p* < 0.01) that provide functional insights into the coordinated metabolic and transcriptional changes under salt stress. The strong positive correlation between oxaloacetate and genes encoding key enzymes such as phosphoenolpyruvate carboxykinase (PCKA, PCK1) and citrate synthase (CSY2) suggests coordinated upregulation consistent with enhanced activity in anaplerotic pathways that replenish the TCA cycle, thereby potentially sustaining carbon flux through central metabolism [32]. In contrast, genes involved in sucrose and starch biosynthesis (SPS, SBEI) were negatively correlated with oxaloacetate and generally downregulated. Together with the downregulation of cellulose synthase genes, this pattern implies a coordinated redirection of carbon flux away from storage carbohydrates and structural polymer biosynthesis and towards osmoprotectant accumulation and core metabolic maintenance. This shift is consistent with a strategic ‘growth-defense trade-off’, in which carbon resources are preferentially allocated to stress adaptation at the expense of growth-related processes [34,35]. The observed correlations suggest that this trade-off may be governed by a coordinated regulatory network linking transcriptional reprogramming with metabolic flux redistribution. Collectively, these findings support a model in which central carbon metabolism may serve as a regulatory hub that helps mediate the balance between growth and defense, thereby influencing the formation of medicinal quality under salt stress.

### 4.4. Implications for the Medicinal Quality of A. roxburghii and Future Prospects

The metabolic reprogramming induced by salt stress has direct and significant implications for the medicinal quality of *A. roxburghii*. The enhanced central carbon metabolism increases the availability of key precursors, such as phosphoenolpyruvate and erythrose-4-phosphate, which serve as entry points for the shikimate and phenylpropanoid pathways responsible for the biosynthesis of flavonoids and other phenolic compounds. Concurrently, the suppression of competing pathways, including cellulose synthesis and structural polysaccharide biosynthesis, suggests a coordinated redirection of carbon flux toward secondary metabolism. This carbon allocation strategy provides a physiological and molecular explanation for the observed enhancement of bioactive compounds under moderate salt stress. Similar stress-induced accumulation of specialized metabolites has been widely reported in medicinal plants [36].

Importantly, these findings extend beyond mechanistic insights into salt tolerance responses. They highlight the potential application of controlled “eustress” (beneficial stress) as a sustainable cultivation strategy to optimize metabolic output. Specifically, moderate salt stress could be strategically applied to redirect carbon flux toward the production of high-value phytochemicals, thereby enhancing both the medicinal quality and economic value of *A. roxburghii*.

### 4.5. Limitations and Technical Considerations

While this study provides insights into the metabolic and transcriptional reprogramming of *A. roxburghii* under salt stress, several limitations should be acknowledged. First, although three biological replicates per treatment are commonly used in targeted metabolomic and transcriptomic studies under controlled conditions, increasing the replicate number would enhance the statistical power and robustness of the findings. Second, the use of a single sampling time point after one month of stress captures a steady-state adaptation response but does not reveal the dynamic progression of metabolic and transcriptional changes. Time-series experiments are needed to resolve the temporal sequence of events. Third, the metabolomic coverage was limited; key glycolytic intermediates (e.g., succinate-CoA, isocitric acid) and the redox cofactor NADPH were not detected, which constrains a more precise analysis of flux distribution and cellular redox state. Fourth, the integrative analysis relied on correlation-based networks. Although stringent statistical thresholds (|r| > 0.9, *p* < 0.01) were applied to identify high-confidence associations, these correlations do not establish direct regulatory or causal relationships. Future studies employing genetic manipulation, enzyme activity assays, or stable isotope tracing are warranted to validate the functional roles of the identified genes and metabolites. Finally, the potential improvement in medicinal quality inferred from carbon reallocation remains to be directly quantified through measurements of specific secondary metabolites (e.g., flavonoids, polysaccharides) under the same stress regime. Addressing these points in future work will help to construct a more mechanistic and dynamic understanding of how salt stress reshapes central carbon metabolism and influences medicinal compound accumulation in *A. roxburghii*.

## 5. Conclusions

This integrated study demonstrates that salt stress induces a coordinated molecular and metabolic reprogramming in *A. roxburghii*. High salinity inhibits growth and photosynthesis, while simultaneously triggering a reconfiguration of central carbon metabolism, characterized by the accumulation of soluble sugars for osmotic adjustment and alterations in energy metabolism. Transcriptomic and metabolomic analyses collectively reveal that carbon flux is redirected from growth-related processes toward osmoprotectant production and stress-responsive pathways. This shift is supported by the up-regulation of glycolysis and TCA cycle genes and the down-regulation of oxidative phosphorylation and polysaccharide biosynthesis. Importantly, this carbon reallocation not only underpins salt tolerance in *A. roxburghii* but also provides a metabolic basis for the potential enhanced accumulation of bioactive secondary metabolites, such as flavonoids, under moderate stress, as it increases the supply of key precursors. The findings highlight the potential of controlled salt stress as a practical cultivation strategy to improve the medicinal quality of *A. roxburghii*.

## Figures and Tables

**Table 1:** Primer used in this study.

GeneBank Accession	Gene	Forward Primer (5′-3′)	Reverse Primer (5′-3′)
PP999134	aconitate hydratase 3	AGGATCGATCGGCTTCCCTA	TCAACCTGCTTTGGTGCAGA
PP973868	phosphoenolpyruvate/phosphate translocator	TGATACGGGATGGTCCTGGT	ACCAAAGCCCAAAAAGCGAG
PP973863	malate dehydrogenase	GGTGCTCAAGCAAAAGGGTG	GCCTCCACCACTTCAGTACC
PP973865	beta-glucosidase 6	TCTCCCACAAGCGACCATTC	TCTGAATCCCCCAAAGCACC
PP973869	glycerate dehydrogenase	ACAGAGTCGTGAGCACCAAG	TGACTCCGTCGCATTTGTCA
PP973866	citrate synthase	ACCCATGGCCGAATGTTGAT	TCCAGCCATTCCATGGTCAC
PP973864	alkaline/neutral invertase	CAGCATGCATAAAGACCGGC	AGAGGCTTCATGGCCTTGTC
PP973860	dihydrolipoyllysine-residue acetyltransferase component 2	TCCAGAGCCGAAGGTTTCAC	CATCGGGGCCAGTACCTTTT
PP999135	Glyceraldehyde-3-phosphate dehydrogenase 2	CGTGTGCCTACAGTGGATGT	TCGTTGTCATACCAGGCCAC
PP973862	pyruvate decarboxylase 1	TCTGCGTGTGAATGTGCTCT	TGACCTGAAAGCTTCCGTCC
PP973867	glycosyltransferase 2	CCGCCGTCATCATCGACTTA	AGAGAGCGGGAAGATGGAGT
PP973861	cellulose synthase-like protein G3	CGGTCATGGCCTACGACTAC	GGGGAGTCGATCATGCTGAG
MH899010	β-actin	AGATGAGGCACAGTCCAAGA	GCTGGAACATTGAAGGTCTC

**Table 2:** Statistics of base composition and sequencing quality.

Sample	CleanData (bp)	AF_Q20 (%)	AF_Q30 (%)	AF_GC (%)	Reads	Total_Mapped (%)
Control-1	7,296,968,063	7,144,959,490 (97.92%)	6,845,610,687 (93.81%)	3,393,464,822 (46.51%)	48,858,088	40,676,273 (83.25%)
Control-2	8,145,348,695	7,978,694,514 (97.95%)	7,646,381,241 (93.87%)	3,765,892,953 (46.23%)	54,433,832	45,406,833 (83.42%)
Control-3	7,939,423,333	7,755,877,487 (97.69%)	7,406,460,578 (93.29%)	3,694,678,830 (46.54%)	53,067,272	44,604,371 (84.05%)
NaCl_50-1	8,046,778,331	7,848,951,235 (97.54%)	7,477,119,163 (92.92%)	3,715,520,221 (46.17%)	53,881,786	44,879,929 (83.29%)
NaCl_50-2	7,251,247,454	7,099,570,555 (97.91%)	6,797,612,034 (93.74%)	3,339,569,259 (46.06%)	48,500,784	40,500,305 (83.50%)
NaCl_50-3	6,540,369,191	6,394,727,691 (97.77%)	6,112,573,416 (93.46%)	2,999,935,404 (45.87%)	43,696,988	36,584,923 (83.72%)
NaCl_100-1	6,102,162,412	5,978,971,536 (97.98%)	5,732,068,701 (93.94%)	2,840,210,058 (46.54%)	40,794,340	34,629,992 (84.89%)
NaCl_100-2	5,447,262,724	5,335,182,784 (97.94%)	5,115,186,646 (93.90%)	2,505,108,258 (45.99%)	36,482,362	30,200,678 (82.78%)
NaCl_100-3	6,521,164,870	6,372,367,799 (97.72%)	6,083,955,954 (93.30%)	3,009,474,750 (46.15%)	43,660,940	36,209,888 (82.93%)
NaCl_200-1	5,737,470,246	5,625,080,399 (98.04%)	5,401,394,117 (94.14%)	2,647,072,533 (46.14%)	38,440,026	31,641,302 (82.31%)
NaCl_200-2	5,986,411,878	5,857,382,189 (97.84%)	5,602,138,421 (93.58%)	2,780,812,286 (46.45%)	40,061,364	33,394,508 (83.36%)
NaCl_200-3	6,053,658,208	5,901,207,857 (97.48%)	5,616,179,179 (92.77%)	2,789,341,942 (46.08%)	40,522,472	33,532,857 (82.75%)

## Data Availability

The datasets presented in this study can be found in online repositories. The names of the repository/repositories and accession number(s) can be found below: http://www.ncbi.nlm.nih.gov/bioproject/1420432 (accessed on 9 February 2026), PRJNA1420432.

## Supplementary Materials

The following supporting information can be downloaded at: https://www.mdpi.com/article/10.3390/genes17050523/s1. Table S1: The determination results of central carbon metabolites under NaCl stress; Table S2: RNA-seq data.

## Abbreviations

The following abbreviations are used in this manuscript:

DEGs	Differentially expressed genes
DAMs	Differentially accumulated metabolites
CCM	Central carbon metabolism
TCA	Tricarboxylic acid cycle
PSII	Photosystem II
QY	Quantum yield
LC-MS/MS	Liquid chromatography-tandem mass spectrometry
UPLC	Ultra Performance LC
ESI	Electrospray ionization
MRM	Multiple reaction monitoring
FDR	False discovery rate
KEGG	Kyoto Encyclopedia of Genes and Genomes
FPKM	Fragments per kilobase of transcript per million mapped reads
qRT-PCR	Quantitative real-time polymerase chain reaction
ANOVA	Analysis of variance
ROS	Reactive oxygen species
GABA	γ-aminobutyric acid
PCKA	Phosphoenolpyruvate carboxykinase A
PCK1	Phosphoenolpyruvate carboxykinase 1
PCK2	Phosphoenolpyruvate carboxykinase 2
PFK3	Phosphofructokinase 3
SPP2	Sucrose-phosphatase 2
MODA	Malic enzyme A
CSY2	Citrate synthase 2
SPS	Sucrose phosphate synthase
SPS1	Sucrose phosphate synthase 1
SPS3	Sucrose phosphate synthase 3
SBEI	Starch branching enzyme I
ATP	Adenosine triphosphate
ADP	Adenosine diphosphate
NADPH	Nicotinamide adenine dinucleotide phosphate
